# Compound Dislocation of the Long Bones Considered with Especial Reference to the Value of Resection

**Published:** 1857-11

**Authors:** 


*Compound Dislocation of the Long Bones considered with especial reference to the value of resection. By Frank Hastings Hamilton, M.D.. Prof. of Surgery in the Medical Department of the University of Buffalo. Extracted from the American Journal of the Med. Sciences for October*, 1857.

This pamphlet contains sixteen pages of paper and volumes of valuable practical matter. Dr. Hamilton's fame is equaling that of his distinguished relative of fiscal renown; his name belongs to the whole scientific world, and we are mistaken if it does not remain in this position while surgery is cultivated as one of the ameliorating and salutary sciences. As will be seen by the title of the pamphlet given above, he is still enthusiastically absorbed in his favorite study—the surgery of the bones. He is one of the few cultivators of science who seldom makes an unprofitable move. Everything he writes is to the point and in the highest degree practical, full, explicit, intelligible and yet concise.

The object aimed at and accomplished is improvement of the practice of his profession. In this paper the subject is handled in his usual manner. He thinks that the danger of compound dislocations does not arise from the inflammation of the synovial membranes of the joints resulting from its exposure, nor to the extensive lacerations of the tendons, ligaments and membranes attaching and binding the bones together, but to circumstances otherwise operating.

He establishes, by calling to his aid the experience of the ablest surgeons of ancient and modern times, the fact that these injuries are more serious in their effects upon the system—indeed, in the large joints generally fatal—when the bones are reduced and dressed than when left to nature. From this and other facts he adduces the opinion that the extensive inflammations which follow reduction and bandaging result from the continued tension of the stretched, lacerated and bruised tendons, muscles and ligaments, acting like strong exercise of function in any organ in keeping up vascular and nervous excitement to such a pitch as to aggravate the inflammation. The inflammation will not subside any more than rheumatic inflammation will subside in a joint or muscle that is constantly exercised. Resection by shortening the limb takes off the fatiguing and irritating tension of the soft parts, and by allowing them to assume a condition of repose, favors in this important manner the natural tendency to resolution. He argues also that if inflammation of the synovial membrane is so much to be dreaded, as some modern writers believe, removal of the articulating ends of the bones is an effectual way of avoiding that evil. Lastly, cases are adduced as experimental evidence in favor of resection, which he thinks terminated more favorably after the operation than they could have done by the ordinary modes of treatment. To properly appreciate his arguments and facts in this kind of treatment of compound dislocations, his paper must be read, which, we hope, every surgeon will not fail to do.

